# Supplementary material for: Hf Doping Boosts the Excellent Activity and Durability of Fe-N-C Catalysts for Oxygen Reduction Reaction and Li-O2 Batteries
Source: Nanomaterials (Basel). 2024 Dec 13;14(24):2003. doi: 10.3390/nano14242003 (PMC11728555; doi:10.3390/nano14242003)
Supplement: Supplementary file 1 [file nanomaterials-14-02003-s001.zip › nanomaterials-3365130-supplementary.pdf]

Hf Doping Boosts the Excellent Activity and Durability of Fe-N-C Catalysts for Oxygen Reduction Reaction and Li-O_2 Batteries

Mingrui Liu [1,*], Shaoqiu Ke [2,*], Chuangqing Sun [3], Chenzhuo Zhang [3] and Shijun Liao [4]

[1] National Energy Key Laboratory for New Hydrogen-Ammonia Energy Technologies, Foshan Xianhu Laboratory, Foshan 528200, China
[2] Hubei Key Laboratory of Photoelectric Materials and Devices, School of Materials Science and Engineering, Hubei Normal University, Huangshi 435002, China
[3] State Key Laboratory of Advanced Technology for Materials Synthesis and Processing, Wuhan University of Technology, Wuhan 430070, China; scq11@whut.edu.cn (C.S.); 18271774069@163.com (C.Z.)
[4] The Key Laboratory of Fuel Cell Technology of Guangdong Province, School of Chemistry and Chemical Engineering, South China University of Technology, Guangzhou 510641, China; chsjliao@scut.edu.cn
* Correspondence: liumingrui@xhlab.cn (M.L.); shaoqiuke@hbnu.edu.cn (S.K.)

S1. Experimental section

Materials

Zinc nitrate hexahydrate ($Zn(NO_3)_2·6H_2O$), 2-methylimidazole, hafnium acetylacetonate ($C_{20}H_{28}HfO_8$), ferrocene, methanol, ethanol, Isopropyl alcohol and hydrofluoric acid (HF) were purchased from Aladdin. Commercial Pt/C (40 wt.%, HiSPEC4000) was purchased from Alfa Aesar, and Nafion dispersion (5 wt.%) was obtained by diluting a Nafion D2021 dispersion. All reagents were used as received.

Sample preparation

Preparation of precursor ZIF-8 and ZIF-8-Hf: Firstly, 6.0 g 2-methylimidazole and 40 mL anhydrous methanol were blended with a magnetic stirrer to obtain a evenly dispersed mixture, named as mixture A. Secondly, 1.6 g $Zn(NO_3)_2·6H_2O$ and 20 mL anhydrous methanol were blended with a magnetic stirrer to obtain a evenly dispersed mixture, named as mixture B. Thirdly, the mixture B was slowly poured into mixture A, stirred at room temperature for 12 h, centrifuged by high-speed centrifuge, and washed with anhydrous methanol several times. Lastly, the collected white samples were dried in a vacuum oven at 333 K for 8 h to yield catalyst precursor ZIF-8; the synthesis process of the precursor Hf@ZIF-8 is an improvement of ZIF-8: the mixture A was replaced by 6.0 g 2-methylimidazole with different mass of $C_{20}H_{28}HfO_8$, respectively. A series of Hf-ZIF-8 with different Zn/Hf mass ratios were named as Hf-ZIF-8-x/y, where x/y is the mass ratio of Hf to Zn.

Synthesis of catalysts: The Fe-Hf/N/C and Fe/N/C catalysts were synthesized via a high-temperature pyrolysis method combined with a gaseous-doping approach. Firstly, ferrocene was utilized as the Fe source and placed at one end of a quartz boat, while the MOFs material (Hf-ZIF-8-x/y or pure ZIF-8) was loaded at the other end. Another quartz boat was then used to cover the first. The quartz boats were introduced into a tubular furnace with a high-purity nitrogen flow. The materials were heated at 423 K for 2 h to facilitate the evaporation of ferrocene, allowing the vaporized ferrocene molecules to enter the micropores of ZIF-8 or Hf-ZIF-8-x/y. Subsequently, the system was pyrolyzed/doped at 1173 K for 3 h. The followed by treatment with a 0.5 M HF solution at 353 K for 4 h to remove inactive metal compounds. After acid leaching, the samples were washed several times with deionized water. Finally, the samples were heated to 1173 K at a rate of 10 K min^{-1} in a nitrogen atmosphere and held at this temperature for 1 h to further promote graphitization. Moreover, Hf/N/C and N/C catalysts were prepared in the same manner but without the addition of ferrocene during pyrolysis. The obtained catalysts are named as Fe-Hf/N/C-x/y-a/b-T, where x/y represents the mass ratio of hafnium acetylacetonate in the precursors, a/b represents the mass ratio of precursors to ferrocene, and *T* represents the pyrolysis temperature. It is worth

noting that the mass ratio a/b was set within the range of [9:11-9:15]. For instance, in the synthesis of some of the most representative samples, we used approximately 0.5 g of the MOFs material (Hf-ZIF-8 or ZIF-8) and 0.62-0.83 g of ferrocene. This ratio was optimized through a series of preliminary experiments to ensure an appropriate amount of Fe doping while maintaining the structural integrity and catalytic activity of the catalysts.

Preparation of MEAs: Catalyst-coated membranes (CCM) were fabricated by spraying catalyst inks onto a membrane. JM 40 wt.% Pt/C was employed as the anode catalyst, while Fe-Hf/N/C or Fe/N/C was used as the cathode catalyst. Nafion 211 membrane (Dupont, USA) served as the proton exchange membrane (PEM). The Pt loading in the anode catalyst layer was 0.1 mg cm^{-2}. The loadings of Fe-Hf/N/C and Fe/N/C in the cathode catalyst layer were 0.5, 1.0, 1.5, and 2.0 mg cm^{-2}, respectively. The dry Nafion content in the anode and cathode catalyst layers was 30 and 50 wt.%, respectively. The gas diffusion layers (GDL) were prepared by immersing carbon paper in a Teflon solution, followed by drying, calcining, and spraying a carbon slurry (carbon powder (XC-72) and PTFE) onto the carbon paper to form a microporous layer (MPL). The loading of carbon powder in the MPL was 3 mg cm^{-2}, and the PTFE content was 20 wt.%. The CCM and GDL were then pressed together to form the membrane electrode assembly (MEA).

Characterization

The phase constituents of all samples were analyzed using an X-ray diffractometer (XRD, TD-3500, Tongda, China) with Cu-Kα radiation and Fourier transform infrared spectroscopy (FTIR) with the Equinox 55 (Bruker, Germany). The morphologies of the samples were characterized by an SU-8200 scanning electronic microscope (SEM, Hitachi, Japan). Transmission electron microscopy (TEM) was performed using a JEM-2100HR (JEOL, Japan) equipped with an EDS detector at an operating voltage of 200 kV. X-ray photoelectron spectroscopy (XPS) was carried out on an ESCALAB 250 (Thermo-VG Scientific). X-ray photoelectron spectroscopy (XPS) was conducted on an ESCALAB 250 (Thermo-VG Scientific). A Tristar 3020 gas adsorption analyzer (Micromeritics, USA) was used to measure the specific surface area and pore size distributions. The X-ray absorption find structure spectra (Fe K-edge) were collected at 1W1B station in Beijing Synchrotron Radiation Facility (BSRF). The storage rings of BSRF were operated at 2.5 GeV with an average current of 250 mA. Using Si(111) double-crystal monochromator, the data collection were carried out in transmission/fluorescence mode using ionization chamber. All spectra were collected in ambient conditions.

XAFS Analysis and Results

The acquired EXAFS data were processed according to the standard procedures using the ATHENA module implemented in the IFEFFIT software packages. The k^3-weighted EXAFS spectra were obtained by subtracting

the post-edge background from the overall absorption and then normalizing with respect to the edge-jump step. Subsequently, k^3-weighted $\chi(k)$ data of Fe K-edge were Fourier transformed to real (R) space using a hanning windows (dk=1.0 Å-1) to separate the EXAFS contributions from different coordination shells. To obtain the quantitative structural parameters around central atoms, least-squares curve parameter fitting was performed using the ARTEMIS module of IFEFFIT software packages.

Properties measurement

An Autolab electrochemical workstation (Metrohm, Netherlands) with a standard three-electrode system was employed to measure the electrochemical performance of the catalysts. In an acidic medium, Ag/AgCl and a carbon rod were used as the reference electrode and counter electrode, respectively. The working electrode was an RDE or RRDE coated with a catalyst film. Typically, 5 mg of Fe-Hf/N/C or Fe/N/C was dispersed in 1 mL of a Nafion/isopropanol solution (0.25 wt.% Nafion) by sonication, and then 20 µL of the catalyst ink was coated onto an RDE or RRDE. 40 wt.% Pt/C, purchased from Johnson Matthey, was selected for comparison.

All linear sweep voltammetry (LSV) measurements of the oxygen reduction reaction (ORR) were recorded at a scan rate of 5 mV s^{-1} and 900 rpm in O_2-saturated 0.1 M $HClO_4$ solution. Cyclic voltammograms (CV) were obtained at scan rates ranging from 5 to 25 mV s^{-1} in N_2-saturated 0.1 M $HClO_4$ solution. The hydrogen peroxide yield (H_2O_2%) was calculated using the equation: H_2O_2 (%) = $(200I_{ring}/N)/(I_{disk}+(I_{ring}/N))$, where I_{ring} and I_{disk} are the absolute values of the ring and disk current, respectively, and N is the collection efficiency at the ring electrode (N = 0.37). The stability of the catalysts in an RDE was tested using chronoamperometry with a constant voltage of 0.62 V for 48,000 s (at 900 rpm) in a solution of O_2-saturated 0.1 M $HClO_4$.

Single PEM fuel cell testing

The MEAs were assembled in a PEM fuel cell with an active area of 5 cm^2 and supplied with hydrogen (99.999%, 300 mL min^{-1}), oxygen (99.999%, 300 mL min^{-1}), or compressed air (600 mL min^{-1}). Each PEM fuel cell with MEAs was tested in a Fuel Cell Test System (Arbin Instruments, USA). Operating conditions: the battery temperature was maintained at 353 K, the back pressure of anode and cathode reactant gases was 150 or 200 kPa (table value), and the relative humidity of the reactant gases was 100%. The stability and durability of MEAs with Fe-Hf/N/C and Fe/N/C as cathode catalysts were evaluated using the constant current discharge method.

Li-O_2 Battery Measurements

The CR2032 coin cell with holes opened cathode was used for the performance measurements. The cathode catalyst slurry was prepared by mixing our prepared catalyst with 5% poly (vinylidene fluoride) as the bonding

agent in a mass ratio of 8:2. The prepared slurry was then spread onto pretreated carbon paper with a mass loading of about 0.3 mg cm^{-2} and dried in a vacuum for 24 h. The Li-O_2 battery was assembled in an argon-filled glovebox, with catalyst-coated carbon paper as the cathode, a piece of Ni foam as the cathode support, 1.0 M LITFSI (in tetraethylene glycoldimethyl ether (TEGDME)) as the electrolyte, a glass fiber as the separator, and a fresh Li foil as the anode. Electrochemical measurements were conducted on an Ivium electrochemical workstation, and the discharge‑charge performance measurements were performed using a Neware Battery Testing System (Shenzhen, China) under 1 bar O_2 pressure. All the specific capacities were calculated using the weight of the catalyst loaded on the air cathode.

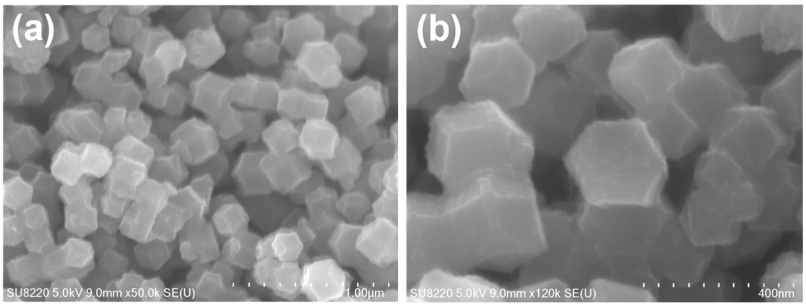

Supplementary Figure S1 SEM image of Fe-/N/C-2/400 (**a**) and (**b**).

S2. BET analysis of catalyst

The N_2 adsorption/desorption was used to test the specific surface areas of four catalysts, the N/C, Hf/N/C, Fe/N/C and Fe-HF /N/C, and their N_2 adsorption/desorption curves and specific surface areas were shown in **SFig. 2**. The specific surface areas of the four catalysts are 1162.04, 1287.78, 973.02 and 994.37 m^2 g^{-1}, respectively. It can be seen from the comparison that the specific surface area of Hf/N/C is larger than that of the N/C, while that of the Fe-Hf/N/C is larger than that of the Fe/N/C. It is shown that the specific surface area of the catalyst can be increased by the introduction of Hf. The increase of specific surface area of target catalyst Fe-Hf/N/C is conducive to the dispersion of active sites on the catalyst surface and the improvement of catalytic activity of the catalyst.

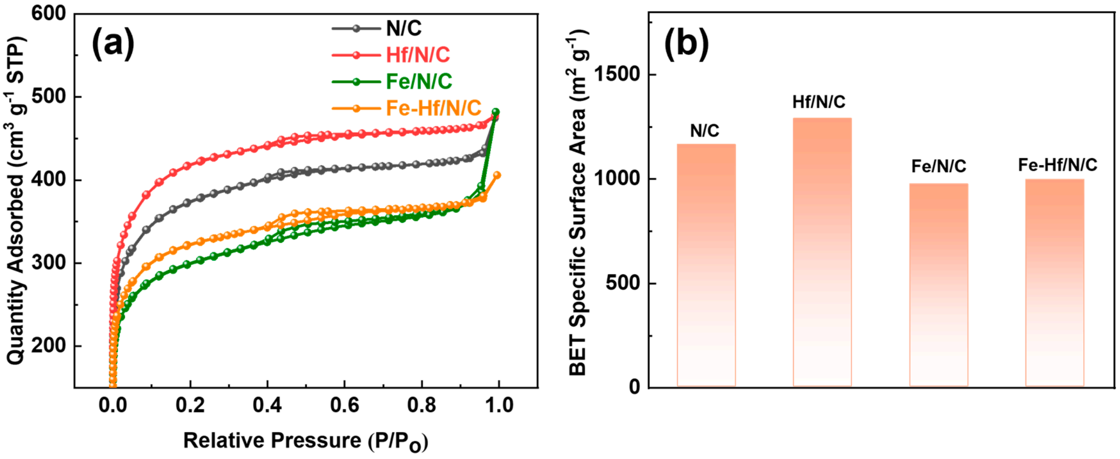

Supplementary Figure S2 BET analysis. The N_2 adsorption and desorption isotherms (**a**) and BET specific surface area (**b**) of N/C, Hf/N/C, Fe/N/C and Fe-Hf/N/C catalysts.

S3. XAFS analysis

The EXAFS fitting results of Fe-Hf/N/C catalysts can better understand the Fe coordination environment. From the fitting results, the average coordination number of Fe-N can be obtained as 4.7 (**STable 1**), indicating that Fe-N coordination in the catalyst is mainly a mixture of Fe-N_4 and Fe-N_5. The fitting results show that Fe is atomically dispersed in the catalyst to form a coordination structure of FeN_4/FeN_5.

Supplementary Table S1 EXAFS fitting parameters at the Fe K-edge for various samples

Sample	Shell	N [a]	R (Å) [b]	σ^2 (Å^2·10^{-3}) [c]	ΔE0 (eV) [d]	*R* factor (%)
**Fe/N/C**	Fe-N	4.5	1.96	8.7	-2.3	0.8
**Fe-Hf/N/C**	Fe-N	4.7	1.98	8.6	-1.1	0.3

[a] *N*: coordination numbers; [b] *R*: bond distance; [c] σ^2: Debye-Waller factors; [d] ΔE_0: the inner potential correction. *R* factor: goodness of fit. *S*02 was set as 0.87 for Fe-N, which was obtained from the experimental EXAFS fit of reference FePc by fixing CN as the known crystallographic value and was fixed to all the samples. (Note: The error range of N, σ^2 is 20%, and the accuracy range of R is ±0.03 A.)

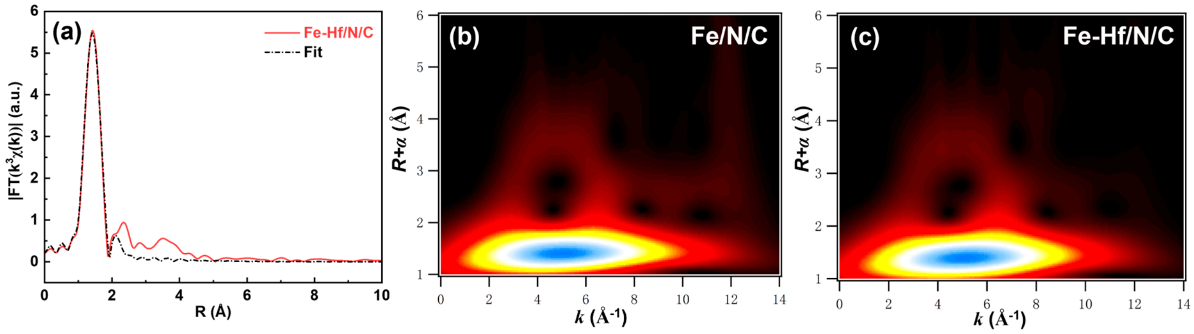

Supplementary Figure S3 XAFS Analysis. (**a**) Fe Fourier transformed spectra EXAFS spectrum and fitting spectra of Fe-Hf/N/C. Wavelet transforms for the k^3-weighted Ru K-edge EXAFS signals of (**b**) Fe/N/C and (**c**) Fe-Hf/N/C.

S4. X-ray photoelectron spectroscopy (XPS) analysis

The relative atomic ratios of the relevant elements were obtained by the XPS survey spectra and high-resolution spectra (**Table S2**). For the Fe-Hf/N/C sample, the atomic percentages of C 1s, Fe 2p, Hf 4f, and N 1s are 93.18%, 0.62%, 0.25%, and 5.95%, respectively. In the Fe/N/C sample, the atomic percentages of C 1s, Fe 2p, and N 1s are 96.17%, 0.3%, and 3.53%, respectively. For the Hf/N/C sample, the atomic percentages of C 1s, Hf 4f, and N 1s are 94.47%, 0.2%, and 5.33%, respectively. These XPS results clearly demonstrate the presence of C, Fe, Hf, and N elements in our samples, which is consistent with the expected elemental composition of the catalysts. The relative atomic ratios obtained from XPS provide quantitative evidence for the existence of these elements and support the conclusions drawn from our other characterization techniques.

Supplementary Table S2 The relative atomic ratios of the relevant elements were obtained by the XPS survey spectra and high-resolution spectra.

**Nos**	**C1s**	**Fe2p**	**Hf4f**	**N1s**	**Atomic %**
**Fe-Hf/N/C**	93.18	0.62	0.25	5.95	100
**Fe/N/C**	96.17	0.3		3.53	100
**Hf/N/C**	94.47		0.2	5.33	100
